# Real-World Treatment Patterns, Clinical Outcomes, Healthcare Resource Utilization, and Costs in Advanced Hepatocellular Carcinoma in Ontario, Canada

**DOI:** 10.3390/cancers16122232

**Published:** 2024-06-15

**Authors:** Soo Jin Seung, Hasnain Saherawala, YongJin Kim, Jimmy Tieu, Sharon Wang, Cal Shephard, Dominick Bossé

**Affiliations:** 1 HOPE Research Centre, Sunnybrook Research Institute, 2075 Bayview Avenue, Toronto, ON M4N 3M5, Canada; saherawala.hasnain@gmail.com; 2 AstraZeneca Canada, 1004 Middlegate Road, Mississauga, ON L4Y 1M4, Canada; yongjin.kim@astrazeneca.com (Y.K.); jimmy.tieu@astrazeneca.com (J.T.); sharon.wang@astrazeneca.com (S.W.); cal.shephard@astrazeneca.com (C.S.); 3 Department of Medicine, University of Ottawa, 501 Smyth Road, Ottawa, ON K1H 8L6, Canada; dbosse@toh.ca

**Keywords:** advanced hepatocellular carcinoma, HCC, survival, costing, resource utilization, administrative data, real-world evidence, Canada

## Abstract

**Simple Summary:**

Sorafenib has been the standard of care for advanced hepatocellular carcinoma (aHCC) patients since 2008. Recently, new therapies have been funded that provide increased survival in this patient population. The aim of our retrospective cohort study of linked administrative databases in Ontario was to understand the characteristics of patients with aHCC, their treatment patterns, survival outcomes, healthcare resource utilization (HCRU), and costs. We observed that patients who received systemic therapy for aHCC had a higher median overall survival (mOS) from diagnosis compared to patients who received other systemic therapies or a locoregional treatment (LRT). The mean cost per patient was $52,166 CAD for all the patients in the study, with inpatient hospitalizations and oral medications as the largest cost drivers.

**Abstract:**

The therapeutic landscape for aHCC has evolved in recent years, necessitating a comprehensive analysis of treatment patterns, clinical outcomes, HCRU, and costs to contextualize emerging treatments. This study aimed to investigate these outcomes using real-world data from Ontario, Canada. This retrospective cohort study was conducted using linked administrative databases from April 2010 to March 2020. Patients diagnosed with aHCC were included, and their clinical and demographic characteristics were analyzed, as well as treatment patterns, survival, HCRU, and economic burden. Among 7322 identified patients, 802 aHCC patients met the eligibility criteria for inclusion in the study. Treatment subgroups included 1L systemic therapy (53.2%), other systemic treatments (4.5%), LRT (9.0%), and no treatment (33.3%). The median age was 66 years, and the majority were male (82%). The mOS for the entire cohort from diagnosis was 6.5 months. However, patients who received 1L systemic therapy had an mOS of 9.0 months, which was significantly higher than the other three subgroups. The mean cost per aHCC-treated patient was $49,640 CAD, with oral medications and inpatient hospitalizations as the largest cost drivers. The results underscore the need for the continuous evaluation and optimization of HCC management strategies in the era of evolving therapeutic options.

## 1. Introduction

Hepatocellular carcinoma (HCC) is a primary liver cancer associated with poor survival outcomes. In 2022, it was estimated that 3500 patients were diagnosed with HCC, and 1650 patients died from it in Canada [1]. The Barcelona Clinic Liver Cancer (BCLC) staging system classifies HCC as stage 0, A, B, C, and D, corresponding to very early, early, intermediate, advanced, and terminal stage, respectively [2]. One study reported that 7% of North Americans (United States and Canada) had stage 0 at diagnosis, while 30% had stage A, 10% had stage B, 42% had stage C, and 11% had stage D [3].

Systemic therapy is recommended for patients diagnosed with advanced HCC (aHCC) and intermediate HCC and generally not amenable to locoregional treatment (LRT) [4,5]. Sorafenib was the only Health Canada-approved first-line (1L) therapy for aHCC between 2008 to 2020 [6]. However, a number of therapies have considerably changed the therapeutic landscape, including lenvatinib (2020) [7], atezolizumab in combination with bevacizumab (2022) [8,9,10], and tremelimumab in combination with durvalumab (2023) [11]. In second-line (2L) and beyond, regorafenib and cabozantinib are commonly used [12,13].

Limited contemporaneous evidence is available in the real-world setting, including treatment patterns, clinical outcomes, healthcare resource utilization (HCRU), and costs associated with the management of aHCC in Canada. One study reported an mOS of 9.2 months (95% CI: 8.0–10.4 months), which included 643 HCC patients across Canada (British Columbia, Alberta, and Ontario) who received sorafenib between January 2008 and June 2015 [14]. Another study identified 2297 HCC patients in Ontario between 2005 and 2009, which reported a 1-year relative survival of 54% and a 5-year survival rate of 20% [15]. There is a dearth of evidence available on HCRU and costs associated with aHCC in Canada, with one study that calculated the mean (95% CI) 5-year net costs of HCC care as $77,509 CAD in 2010 but did not demonstrate HCRU and costs stratified for the aHCC patients [16].

In light of the evolving therapeutic landscape for HCC in recent years, there is a need for studies to scrutinize the effectiveness of systemic therapy and the impact on HCRU during the sorafenib era. This is essential for establishing benchmarks and enhancing our comprehension of novel treatments post-sorafenib. This study aims to use real-world, population-level data from databases available in Ontario, Canada to understand treatment patterns and the clinical and economic burdens in the aHCC patient population.

## 2. Materials and Methods

### 2.1. Overall Study Design

This study was a retrospective cohort study of linked administrative databases available from the Institute for Clinical Evaluative Sciences (ICES) in Ontario. Patients diagnosed with aHCC between 1 April 2010 and 31 March 2019 were analyzed (post-31 March 2020 period was avoided due to the impact that the COVID-19 pandemic had on cancer management). Patients were followed until the last day of available follow-up, date of death, or 31 March 2020, whichever occurred first.

### 2.2. Data Sources

Data were accessed through ICES, which collects data on public coverage via the Ontario Health Insurance Plan (OHIP) and other population-level health information. To determine the trajectory of care over time, health information on each individual patient was linked to applicable datasets. The following databases were used: the Ontario Cancer Registry (OCR) for diagnoses, New Drug Funding Program (NDFP), Activity Level Reporting (ALR) and Ontario Drug Benefit (ODB) Program for medication use, the National Ambulatory Care Reporting System (NACRS) for cancer clinic and emergency room visits, the Ontario Laboratories Information System (OLIS) for laboratory information from community labs, the Discharge Abstract Database (DAD) for inpatient hospitalizations and same-day surgeries, Ontario Health Insurance Plan (OHIP) for physician billing, laboratory claims, and non-medical specialties data, and the Office of the Registrar General-Deaths (ORGD) for vital statistics. Other databases, such as complex continuing care and home care, were also used.

### 2.3. Cohort Definition

ICD-10 codes C22.0, C22.4, C22.8, and C22.9, as well as ICD-O-3 code C22.0 (with morphology codes 81703, 81713, 81723, 81733, 81743, and 81753) were used to identify patients diagnosed with HCC. In the absence of BCLC and Child–Pugh status in administrative databases, collaborative staging (stages I-IV, unknown, or missing) and receipt of systemic treatment were used to classify aHCC patients. All stage IV patients were categorized as aHCC patients, regardless of treatment status. In addition, patients were categorized as advanced if they were diagnosed with stage I-III or with an unknown or missing stage, who received systemic therapy as their first treatment after diagnosis. Stage I-III or unknown- or missing-stage patients who did not receive any treatment post-diagnosis or received initial treatments other than systemic therapy (e.g., ablation, embolization, surgery) were excluded.

In Table 1, the overall cohort was split into 4 subgroups: (1) patients who received 1L HCC-specific therapy referred to as the “1L systemic” group; (2) patients who received systemic therapy that was not HCC-specific (such as platinum-based chemotherapies), categorized as the “other systemic treatment” group; (3) stage IV patients who received no systemic therapy but did receive LRT, referred to as the “LRT” group; and (4) patients who received no active treatment, or treatment of any kind, following diagnosis, referred to as the “no treatment” group. The index dates for the treated patients were set to the date of treatment initiation (subgroups 1–3). The index date for patients that received no treatment at all (subgroup 4) was the diagnosis date.

Patients were excluded from our study if they were under the age of 18 years or over 105 years as of the index date, were not a resident of Ontario, did not have a valid ICES Key Number, which would prevent them from being linked to ICES data, had a gap in OHIP coverage of 90 days or more in the 2 years prior to the index date, had HCC diagnosis on or after their date of death, or if they had a history of diagnosis of other cancers (except non-melanoma skin carcinoma, cervical carcinoma in situ, or ductal carcinoma in situ, unless they have been in remission for 5 years or more).

### 2.4. Methodology and Clinical Outcomes

The primary outcome of interest was overall survival (OS). OS was reported from either the date of diagnosis (for all subgroups) or treatment initiation (limited to treated patients; subgroups 1–3) until death from any cause or being lost to follow-up, with mean (including standard deviation [SD]), median (including corresponding 95% CI), and interquartile range (IQR) estimated using the Kaplan–Meier methods. Patients not known to have died at the time of the analysis were censored based on the last recorded date the patient was known to be alive. OS was reported for each of the subgroups within the subgroups described above.

Demographic and clinical characteristics of aHCC patients were summarized descriptively. The number of patients with missing values were reported, and no imputation was performed. Patient demographics were determined on the date of diagnosis. Comorbidities were determined through a 2-year lookback window prior to the diagnosis date. Prior disease information (i.e., HBV, HCV, NAFLD, cirrhosis, alcoholic cirrhosis, admission for cirrhosis decompensation) was determined from 4 years prior to the diagnosis date. Prior treatment information was determined from the diagnosis date until the index date (i.e., date of treatment initiation) for the subgroups that were treated.

Treatment pattern tables were generated to determine the relative sample sizes and proportions of patients receiving different therapies along the various treatment trajectories from first-line (1L) to subsequent lines of treatment.

### 2.5. Economic Outcomes

An economic analysis was conducted to understand the HCRU and associated costs for patients with aHCC. HCRU and costs were summarized and reported as the overall total and mean utilization/cost per patient. The mean cost per patient was calculated by dividing the cohort’s total cost by the number of patients alive and eligible at the start of a given year. Costs were reported in 2021 Canadian dollars using a macro-based costing methodology called GETCOST, available from ICES [17]. For total cost, the macro was programmed to determine the costs of short-term episodes (for example, hospital-based encounters) by multiplying the encounter resource intensity weight by an annual cost per weighted case. Long-term episode costs (for example, complex continuing care) were calculated by weighted days, and costs of visit-based encounters were determined at utilization (a bottom-up approach). This costing methodology was described in a previous publication [18,19]. Costs for the cohorts included costing data up to death, end of OHIP eligibility, or end of follow-up.

## 3. Results

### 3.1. Baseline Characteristics

**Figure 1:**
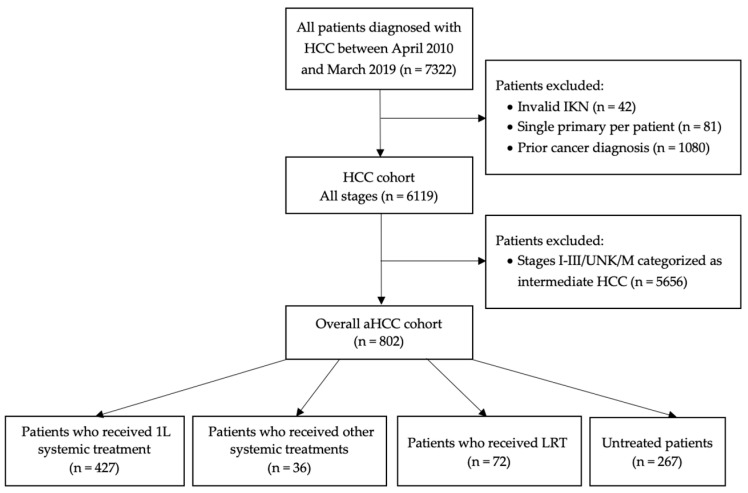
Study patient diagram.

A total of 7322 HCC patients were identified at the start of the cohort definition, of which 802 met the eligibility criteria and were included in the study cohort (see Figure 1). A total of 427 (53.2%) patients received 1L systemic treatment (subgroup 1), 36 (4.5%) received other systemic treatments, such as platinum-based chemotherapies (subgroup 2), 72 (9.0%) did not receive a systemic therapy but received an LRT (subgroup 3), and 267 patients (33.3%) did not receive any treatment at all (subgroup 4).

Table 2 provides the baseline characteristics of the overall cohort. The median age of the overall cohort was 66 years, with the 1L systemic subgroup being older than the other three subgroups. A total of 82% of patients were male, and the mean Charlson score ranged from 1.8–2.2. Patients had a mean duration of disease (diagnosis until end of follow-up) of 1.0 year. Patients who received no treatment (subgroup 4) had a numerically shorter duration of disease than the other three subgroups.

### 3.2. Treatment Patterns

A total of 427 patients received 1L HCC-specific systemic therapy for a median duration of 2.4 months, with the majority (n = 418; 98%) having received sorafenib for 1L treatment (Table 3). Only 4.4% of patients (n = 19) went on to receive 2L systemic therapy, with the majority receiving regorafenib. Of the 72 patients who received an LRT but not 1L systemic therapy (median time from diagnosis to start of LRT was 2.4 months), the majority were transarterial chemoembolization (TACE) (51.4%) or stereotactic body radiation therapy (SBRT) (45.8%).

### 3.3. Survival Outcomes

**Figure 2:**
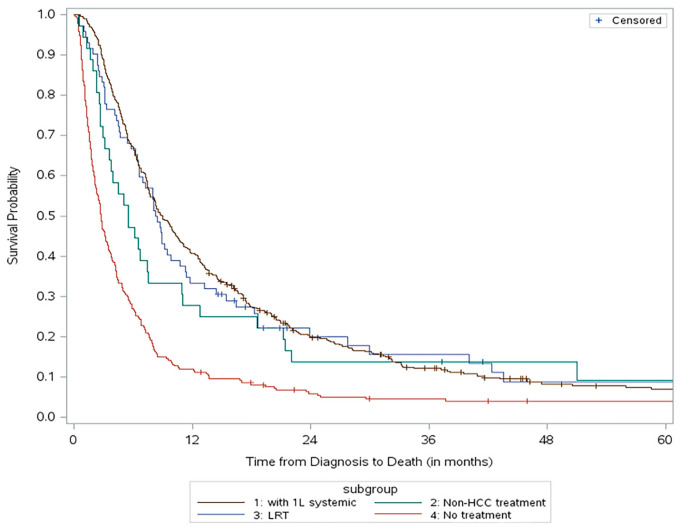
Overall survival from diagnosis to death for all 4 subgroups.

The median OS (mOS) for the overall cohort from diagnosis was 6.5 months (95% CI: 5.8 to 7.2 months). Patients who received 1L HCC-specific systemic therapy had an mOS of 9.0 months (95% CI: 7.8 to 10.3 months) from diagnosis; patients who received other 1L systemic therapies had an mOS of 5.5 months (95% CI: 3.1 to 7.5 months); patients who received only LRT had an mOS of 8.4 months (95% CI: 6.6 to 9.8 months); and patients who received no treatment had an mOS of only 2.7 months from diagnosis (95% CI: 2.3 to 3.3 months) (Figure 2).

**Figure 3:**
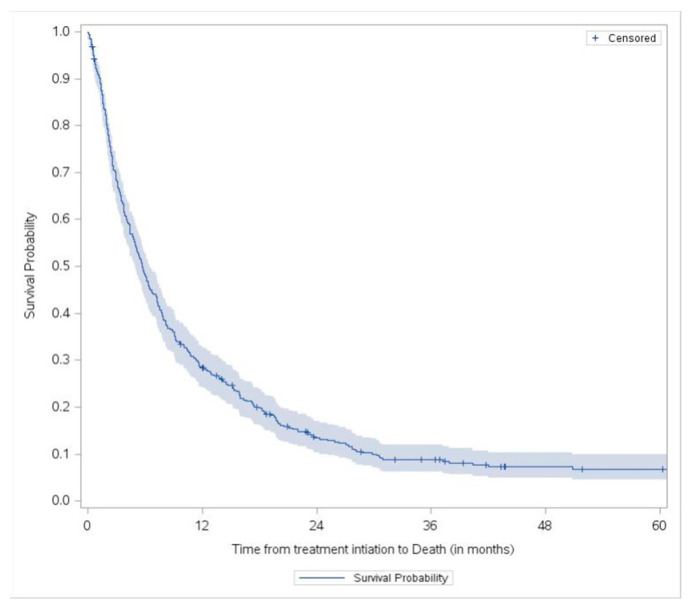
Overall survival from treatment initiation to death for 1L sorafenib-treated patients.

In Figure 3, the 418 patients who received 1L sorafenib had an mOS from treatment initiation of 5.8 months (5.0 to 6.5 95% CI).

### 3.4. Healthcare Resource Utilization

Table 4 provides the HCRU for the overall cohort (n = 802) from diagnosis to death or end of follow-up. A total of 663 patients had an inpatient hospitalization (mean per year = 2.3 admissions), 782 patients had a hospital outpatient clinic visit (mean per year = 11.6 visits), 714 patients had an emergency department visit (mean per year = 3.5 visits), and 358 patients had cancer clinic visits (mean per year = 6.5 visits), with the latter visit type being treatment-related. All mean numbers of admissions and visits were the highest in year 1 and decreased over time. In terms of physician visits specific to oncology, a total of 530 patients visited a medical oncologist (mean per year = 15.9 visits), and 374 patients visited a radiation oncologist (mean per year = 4.6 visits), with both visit types including consultations, assessments, and follow-up visits. While the mean number of visits to the radiation oncologist was highest in year 1 and declined over time, the mean number of visits to the medical oncologist increased with each subsequent year. For oral medications, 706 patients received prescriptions (mean per year = 74.0), with the mean number of drug prescriptions increasing over time (except in year 4).

Table 5 provides the HCRU for the patients who received 1L HCC-specific systemic therapy (n = 427) from treatment initiation to death or end of follow-up. A total of 290 patients had an inpatient hospitalization (mean per year = 1.9), 387 patients had a hospital outpatient clinic visit (mean per year = 9.8), 346 patients had an emergency department visit (mean per year = 3.3), and 195 patients had a cancer clinic visit (mean per year = 5.7). In terms of physician visits, a total of 297 patients visited a medical oncologist (mean per year = 18.7), and 107 patients visited a radiation oncologist (mean per year = 4.3). In contrast, a trend towards increased visits to the medical oncologist was observed subsequent to year 2. For oral medications, a total of 406 patients received prescriptions (mean per year = 75.9) and was the highest in year 2, but it decreased in subsequent years.

### 3.5. Costing

Table 6 provides the costing analysis for the entire cohort (n = 802) from diagnosis to death or end of follow-up. The overall mean cost per patient was $52,166 CAD, with the largest cost drivers being inpatient hospitalizations, oral drug costs, and OHIP physician visits. For overall total costs, the mean cost per patient was highest in year 1 and decreased steadily over time, while the mean cost per patient for inpatient hospitalizations and oral medications was halved from year 1 to year 2. For oral medications, the mean cost per patient increased from year 1 to year 2.

Table 7 provides the costing analysis for the patients who received 1L HCC-specific systemic therapy (n = 427) from treatment initiation to death or end of follow-up. The overall mean cost per patient was $49,640 CAD, with the largest cost driver being oral drug costs, followed by inpatient hospitalizations and OHIP physician visits. For overall total costs, the mean cost per patient was the highest in year 1 and gradually decreased over time, while the mean cost per patient for inpatient hospitalizations was halved from year 1 to year 2.

## 4. Discussion

A total of 802 patients met the advanced HCC diagnosis criteria for our cohort, which was split into four subgroups. Of the 427 patients who received 1L treatment, 98% received sorafenib. Only 4.4% of patients went on to receive 2L systemic therapy, and possible reasons for the low uptake of 2L treatment may be due to patients not living long enough to receive treatment, refusal of treatment, and recency of approval of 2L treatments, such as regorafenib, in 2018. These treatment findings highlight the limited funded therapy options for aHCC patients during the study period of April 2010 to March 2020.

The mOS of 6.5 months for the overall study population from the date of diagnosis is aligned with previous studies conducted in Canada. An analysis of 320 HCC patients diagnosed between January 2011 and December 2015 from the Canadian province of Manitoba reported an mOS from diagnosis of approximately 7 months [20]. Another study examined 1297 advanced unresectable HCC patients in the Canadian province of Alberta and reported an mOS from diagnosis of 12.23 months; however, their patient population included both recurrent and de novo advanced HCC populations [21]. When examining the mOS of only their de novo population, similar to our study findings, they reported an mOS from diagnosis of less than a year (~10 months) [21]. The phase III SHARP trial on sorafenib survival outcomes demonstrated an mOS of 10.7 months for the treatment group.

Our HCRU results for year 1 of the overall cohort were consistently higher than those reported in Alberta [21]: 1.96 versus 1.05 mean inpatient hospitalizations, 2.85 versus 1.82 mean emergency visits, and 11.65 versus 8.96 mean visits with medical oncologists. This could be attributed to varying practice patterns specific to oncology specialists. It should be noted that cancer clinic visits were mutually exclusive to medical oncologist visits, in which the latter was higher (11.65) and refers to consultations, assessments, and follow-up visits, compared to treatment-related cancer clinic visits (5.16). In our study, the overall mean cost per patient from diagnosis was $52,166 CAD, whereas the mean cost per patient for those patients who received 1L HCC-specific systemic therapy from treatment initiation was $49,640 CAD. This can be understood by the later start time for the latter group, and the subgroup of patients treated with LRT were likely hospitalized for their LRTs. The overall mean cost per patient in year 1 from our study ( $39,345 CAD) was similar to the reported mean cost of HCC treatment in the first year after diagnosis ( $37,979 CAD) in a previously published study by Thein and colleagues [16].

Previous studies have demonstrated that evaluating real-world data (RWD) is valuable for analyzing HCC with various applications [16,20,21]. ICES data have been used extensively to assess treatment patterns, healthcare resource utilization (HCRU), prevalence, and survival outcomes across multiple tumor areas in Ontario, Canada [22,23,24,25]. Population-level estimates yielded from our analysis may be of great interest for clinicians to enhance the understanding of the clinical outcomes of aHCC patients under the current treatment landscape and for healthcare decision makers, providing them with information about the economic impacts of different therapeutic options [21,26,27].

While the use of population-level data to examine the treatment patterns and clinical and economic outcomes among aHCC patients was a strength of this analysis, there were some limitations. First, patients who progressed from the intermediate stage (i.e., received LRTs prior to systemic) were not included in this study. Second, administrative data sources lack granular clinical data that may be relevant to HCC. In the absence of such clinical data, we used the receipt of systemic therapy as a proxy for identifying aHCC patients, due to the absence of BCLC and Child–Pugh status, except for the untreated stage IV patients. This is consistent with other studies that have used proxies to classify cohorts from the ICES databases across various tumor areas [28,29,30]. This may have introduced selection bias, since intermediate patients who may have progressed to an advanced disease but did not receive any systemic therapies were excluded from the current analysis. Third, the ODB database only captured data on prescription medications dispensed to persons eligible for publicly funded drug coverage, including those aged 65 years or above. The ODB does not capture information covered by private insurance or compassionate use programs from manufacturers. Fourth, clinical trial information is not collected consistently in administrative databases and does not list the clinical trial drug name (e.g., “clinical trial” is used more as a drug category). Therefore, all clinical trial regimens were excluded from treatment line sequencing and were not analyzed. Correspondingly, the number of patients excluded due to participation in clinical trials was unable to be estimated. Fifth, treatment patterns could vary by geographic location in other provinces, limiting the generalizability of our study findings; lastly, the utilization of newer treatments, such as atezolizumab + bevacizumab and tremelimumab + durvalumab, were not examined in this study, since they received public funding in Ontario succeeding the study period. Future studies with expanded date ranges will likely identify the evolved treatment patterns and the impact of novel and emerging HCC treatments on survival, HCRU, and costs.

## 5. Conclusions

Our study found that aHCC patients being managed in Ontario, Canada between 2010 to 2020 had limited treatment options, as this was reflected in the $52,166 CAD mean cost per patient and in the one-third of patients who received no treatment at all. The low uptake of 1L systemic therapies and the even lower uptake of subsequent 2L systemic therapies underscore high attrition rates, likely related to the frailty of aHCC patients and limited treatment options. The survival, HCRU, and costing outcomes reported here align with other Canadian studies. It is worth noting that since March 2020, there have been approvals for public funding of newer 1L and 2L aHCC systemic treatments. Further studies are needed to assess the impact of novel treatments on disease management and costs associated with aHCC.

## Figures and Tables

**Table 1:** Subgroup definitions.

Subgroup	Definition
1	Patients who received 1L HCC-specific therapy
2	Patients who received other systemic treatments
3	Stage IV patients who received only LRT
4	Patients who received no treatment

**Table 2:** Baseline characteristics of the advanced HCC cohort (* range given due to 1–5 small cell suppression).

	Overall N = 802	1L Systemic N = 427	Other Systemic Treatments N = 36	LRT N = 72	No Treatment N = 267
Mean ± SD age at index date	65.7 ± 12.0	66.9 ± 10.9	61.3 ± 14.8	63.1 ± 15.2	65.0 ± 12.2
Median (IQR) age at index date	66 (59–74)	68 (61–75)	63 (56–71)	63 (57–73)	64 (57–75)
Female	143 (17.8%)	83 (19.4%)	8 (22.2%)	11 (15.3%)	41(15.4%)
Male	659 (82.2%)	344 (80.6%)	28 (77.8%)	61 (84.7%)	226 (84.7%)
Mean ± SD Charlson Score	1.8 ± 1.8	1.8 ± 1.8	2.2 ± 2.5	1.9 ± 1.8	1.8 ± 1.8
Mean ± SD duration of follow-up (years)	1.0 ± 1.5	1.3 ± 1.4	1.2 ± 1.9	1.3 ± 1.8	0.6 ± 1.3
No TACE; n (%)	741 (92.4%)	* 404–408	* 31–35	35 (48.6%)	267 (100%)
Yes TACE; n (%)	61 (7.6%)	* 19–23	* 1–5	37 (51.4%)	0 (0%)
Mean ± SD number of LRT procedures	2.4 ± 2.1	2.7 ± 2.1	4.8 ± 4.5	2.2 ± 1.9	0 (0%)

TACE = transarterial chemoembolization, LRT= locoregional treatment.

**Table 3:** First-line to second-line treatments of the 1L HCC group (* range given due to 1–5 small cell suppression).

	First Line Therapy					
		Sorafenib N = 418	Lenvatinib N = * 4–8	Immunotherapy N = * 1–5	Other N = * 1–5	Total N = 427
Second Line Therapy	Sorafenib—n (%)	0 (0.0%)	* 1–5	0 (0.0%)	0 (0.0%)	* 1–5
	Immunotherapy—n (%)	* 1–5	* 1–5	0 (0.0%)	0 (0.0%)	* 1–5
	Rego—n (%)	* 12–16	0 (0.0%)	0 (0.0%)	0 (0.0%)	* 14–18
	None—n (%)	401 (95.9%)	* 1–5	* 1–5	* 1–5	408 (95.6%)

**Table 4:** HCRU for the overall cohort from diagnosis.

Type of HCRU	Number of Encounters per Patient per Year All Years	Number of Encounters per Patient per Year Year 1	Number of Encounters per Patient per Year Year 2	Number of Encounters per Patient per Year Year 3	Number of Encounters per Patient per Year Year 4	Number of Encounters per Patient per Year Year 5
Inpatient hospitalization admissions	2.3	2.0	1.9	1.7	1.4	NA
Hospital outpatient clinics	11.6	9.1	7.2	6.3	6.2	6.1
ED visits	3.5	2.9	3.0	2.8	2.2	3.0
Outpatient visits to cancer clinics for treatment	6.5	5.2	5.0	15.7	NA	NA
Oral medication prescriptions	74.0	43.7	66.5	69.9	39.4	60.9
IV chemotherapy drug prescriptions	10.7	9.7	NA	NA	NA	NA
All visits to medical oncologist	15.9	11.7	12.5	14.8	18.3	20.4
All visits to radiation oncologist	4.6	4.2	3.3	3.3	NA	NA
Hepatectomy/Liver Transplant	1.3	1.3	NA	NA	NA	NA
EGD	1.7	1.4	1.4	1.5	NA	NA
TARE	NA	NA	NA	NA	NA	NA
TACE	1.5	1.4	1.3	NA	NA	NA
SBRT	3.9	3.7	3.8	NA	NA	NA
Imaging (CT/MRI/US)	5.7	4.5	3.3	3.9	3.2	2.9

NA = not available due to the value being range (e.g., 1–5) and could not be used in the calculation to determine the number of encounters per patient per year; ED = emergency department; EGD = esophagogastoduodenoscopy; TARE = transarterial radioembolization; TACE = transarterial chemoembolization; SBRT = stereotactic body radiation therapy.

**Table 5:** HCRU for the 1L HCC group from treatment initiation.

Type of HCRU	Number of Encounters per Patient per Year All Years	Number of Encounters per Patient per Year Year 1	Number of Encounters per Patient per Year Year 2	Number of Encounters per Patient per Year Year 3	Number of Encounters per Patient per Year Year 4	Number of Encounters per Patient per Year Year 5
Inpatient hospitalization admissions	1.9	1.7	1.6	2.0	NA	NA
Hospital outpatient clinics	9.8	7.8	6.5	5.7	4.6	6.7
ED visits	3.3	2.7	2.4	3.3	2.8	NA
Outpatient visits to cancer clinics for treatment	5.7	4.8	7.0	NA	NA	NA
Oral medication prescriptions	75.8	44.0	84.4	67.8	38.8	70.0
IV chemotherapy drug prescriptions	NA	NA	NA	NA	NA	NA
All visits to medical oncologist	18.7	14.2	12.9	22.5	19.5	NA
All visits to radiation oncologist	4.3	3.9	3.2	NA	NA	NA
Hepatectomy/Liver Transplant	NA	NA	NA	NA	NA	NA
EGD	1.9	1.7	1.8	NA	NA	NA
TARE	NA	NA	NA	NA	NA	NA
TACE	1.1	1.2	NA	NA	NA	NA
SBRT	4.2	3.8	NA	NA	NA	NA
Imaging (CT/MRI/US)	4.6	3.4	3.2	3.4	3.0	4.0

NA = not available due to the value range (e.g., 1–5) and could not be used in the calculation to determine the number of encounters per patient per year; ED = emergency department; EGD = esophagogastoduodenoscopy; TARE = transarterial radioembolization; TACE = transarterial chemoembolization; SBRT = stereotactic body radiation therapy.

**Table 6:** Costing for the entire cohort from diagnosis.

Costs	Summary	All Years	Year 1	Year 2	Year 3	Year 4	Year 5
	N (patients)	802	802	239	102	62	33
Overall total costs	Mean costs (per patient)	52,166	39,345	25,962	20,671	13,747	14,386
Inpatient hospitalization costs	Mean costs (per patient)	17,988	14,856	6806	5626	3201	1237
Hospital outpatient clinic cost	Mean costs (per patient)	4100	3205	1861	1397	1074	894
ED visits costs	Mean costs (per patient)	1478	1157	668	592	306	267
Outpatient cancer clinic visits cost	Mean costs (per patient)	3023	2097	1483	2643	1023	1038
Oral drug cost	Mean costs (per patient)	12,631	8371	9155	6121	3441	4210
IV chemotherapy drug costs	Mean costs (per patient)	254	127	53	5	284	1409
All OHIP costs (GP + Spec + Shadow billings)	Mean costs (per patient)	8799	6814	3826	3452	2683	2037

ED = emergency department; OHIP = Ontario Health Insurance Plan; GP = general practitioner, Spec = specialist.

**Table 7:** Costing for the 1L HCC group from treatment initiation.

Costs	Summary	All Years	Year 1	Year 2	Year 3	Year 4	Year 5
	N (patients)	427	427	118	46	26	15
Overall total costs	Mean costs (per patient)	49,640	38,764	25,157	20,696	9747	16,954
Inpatient hospitalization costs	Mean costs (per patient)	11,182	9002	4819	6426	1485	1884
Hospital outpatient clinic cost	Mean costs (per patient)	3172	2508	1551	1101	861	1068
ED visits costs	Mean costs (per patient)	1155	890	559	691	294	338
Outpatient cancer clinic visits cost	Mean costs (per patient)	3282	2525	2053	1542	149	130
Oral drug cost	Mean costs (per patient)	20,733	16,150	10,695	7006	2027	10,301
IV chemotherapy drug costs	Mean costs (per patient)	133	133	0	0	0	0
All OHIP costs (GP + Spec + Shadow billings)	Mean costs (per patient)	6790	5159	3603	3409	2075	2238

ED = emergency department; OHIP = Ontario Health Insurance Plan, GP = general practitioner, Spec = specialist.

## Data Availability

The datasets generated and/or analyzed for this study are not publicly available due to Ontario’s Personal Health Information Protection Act (PHIPA). ICES has a prescribed entity designation under the PHIPA, and only their analysts have the necessary approvals to access these datasets.
